# Diffused hepatic angiosarcoma with Kasabach-Merritt syndrome-case report and literature review

**DOI:** 10.1186/s12876-020-01216-z

**Published:** 2020-03-30

**Authors:** Xing-mao Zhang, Yao Tong, Qing Li, Qiang He

**Affiliations:** 1 Department of hepatobiliary surgery, Beijing Chaoyang Hospital, Capital Medical University, 8 Gongti South Street, Chaoyang, 100021 District Beijing China; 2 Department of the Third Thoracic Surgery, Cancer Hospital of Chinese Academy of Medical Sciences, Beijing, China; 3 Department of pathology, Beijing Chaoyang Hospital, Capital Medical University, Beijing, China

**Keywords:** Hepatic angiosarcoma, Kasabach-Merritt syndrome, Liver failure, Prognosis

## Abstract

**Background:**

Hepatic angiosarcoma is a rare malignant tumor featured by highly aggressive behavior and poor prognosis. There are few reports about diffused hepatic angiosarcoma with Kasabach-Merritt syndrome till now.

**Case presentation:**

A male patient with the chief complain of hepatic space-occupying lesion accompanied by disturbance of consciousness and jaundice. Hyperbilirubinemia, anemia, thrombocytopenia, prolonged prothrombin time, hypofibrinogenemia, decreased prothrombin activity, and increased fibrinogen degradation product and D-dimer were confirmed by blood analysis; multiple focal hypodense lesions in liver was detected by abdominal computed tomography. Liver failure and Kasabach-Merritt syndrome induced by hepatic hemangioma was diagnosed before operation and liver transplantation was performed. Hepatic angiosarcoma was finally proven by postoperative pathology. This patient died of tumor metastasis 2 months after operation.

**Conclusions:**

Hepatic angiosarcoma which can generate Kasabach-Merritt syndrome and even liver failure has an extremely poor prognosis; liver transplantation option should not be considered in hepatic angiosarcoma regardless of the reason.

## Background

Hepatic angiosarcoma which is characterized by highly aggressive behavior and rapid progression is a rare tumor that originates from endothelial cells in the liver [1]. It accounts for approximately 0.1–2% of all primary liver malignancies [2]. In adults, diffuse hepatic angiosarcoma is few and far between. Although it was first reported in 1974, there is a paucity of data regarding the diagnosis, treatment and prognosis of this malignancy [3].

Kasabach-Merritt syndrome (KMS), reported by Kasabach and Merritt firstly in 1940, is a rare but potentially life-threatening condition. This syndrome presents as hemolytic anemia, thrombocytopenia, prolonged prothrombin time and hypofibrinogenemia and most often occurs in infants [4]. There have been few reports about KMS induced by diffuse hepatic angiosarcoma till now. Liver failure, induced by pharmacological toxicity and viral hepatitis most frequently, is rarely caused by primary liver tumors or liver metastasis [5]. In this report, a patient who was diagnosed with KMS and liver failure at admission received liver transplantation. Hepatic angiosarcoma was confirmed by pathology postoperatively.

## Case presentation

This study was approved by the Institutional Review Board of Beijing Chaoyang Hospital. A 56-year-old male patient was admitted to our hospital with the chief complaint of disturbance of consciousness and jaundice on Oct. 12, 2018. According to his family’s description, the symptoms of asthenia, epigastric discomfort, nausea and vomiting were generated 2 weeks ago, jaundice of skin and sclera was exhibited about 1 week before referring to local hospital. The patient received laboratory test and CT (Computed Tomography) scan in local hospital. Liver failure induced by hepatic space-occupying lesions was considered, and symptomatic treatment was provided for this patient. Unfortunately, the symptoms got worse during the treatment period, and disturbance of consciousness was developed about 1 week before admitting to our hospital. Further examinations including blood test and multiphase contrast-enhanced CT were conducted when the patient was referred to our center.

**Fig. 1:**
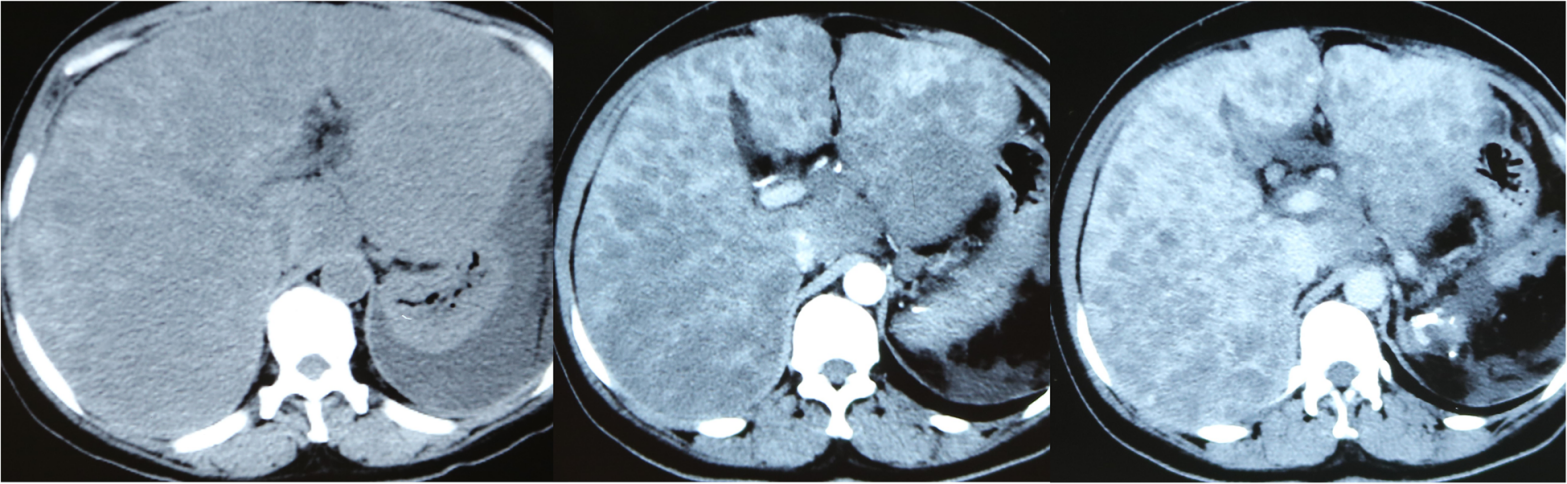
Abdominal computed tomography showed enlarged liver, irregular liver contours, heterogeneous hepatic parenchyma with diffusely distributed multiple hypodense focal lesions

Abnormal laboratory results were as follows: total bilirubin, 449.9 μmol/L (5.0–21.0 μmol/L); direct bilirubin, 280.7 μmol/L (0–6.8 μmol/L); aspartate transaminase, 78 U/L (15-40 U/L); alanine transaminase, 27 U/L (9-50 U/L); ammonia, 204 μmol/L (18-72 μmol/L); albumin, 32.1 g/L (40-55 g/L); white blood cell, 7.28 × 10^9^/L (4.0–10.0 × 10^9^/L); hemoglobin, 67 g/L (120-160 g/L); platelet, 21 × 10^9^ /L (125–350 × 10^9^/L); prothrombin time, 28.3 s (9.6–13.0 s); prothrombin activity, 35.2% (80.0–120.0%); fibrinogen, 81.6 mg/dl (170.0–400.0 mg/dl); fibrinogen degradation product, 74.7 μg/ml (0-5 μg/ml); INR (International Normalized Ratio), 2.19 (0.8–1.2); D-dimer, 25.13 (< 0.55 mg/L FEU); α-fetoprotein, 1.1 ng/mL (< 8.1 ng/mL); carcinoembryonic antigen, 1.0 ng/mL (0–5.0 ng/mL); Carbohydrate Atigen, 19–9 65.5 (0–37 U/mL). Abdominal CT exhibited that the liver was diffusely enlarged and the surface was irregular, multiple focal hypodense liver lesions were distributed diffusely throughout the liver parenchyma (Fig. 1). The patient was diagnosed with liver failure and hepatic hemangioma with KMS based on these results.

**Fig. 2:**
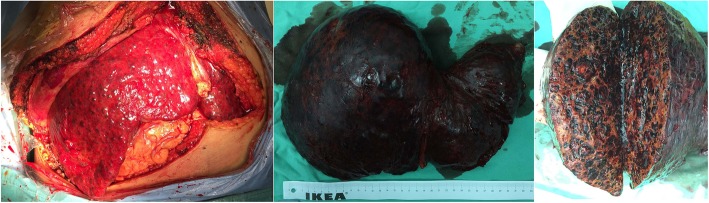
Thickness of left and right liver lobes. The whole liver was infiltrated by tumor, large blood-filled spaces or central necrosis can be seen after cutting open the tumor

Liver transplantation was performed for this patient. Liver came from donation after circulatory death (DCD). Technique of modified piggyback orthotopic liver transplantation was conducted. The enlarged liver could be seen during the operation (Fig. 2). There were no enlarged lymph nodes were detected in the hepatic portal area and hepatoduodenal ligament. Findings including atypical vessels and spindle-shaped cells exhibiting nuclear atypicality were observed under the microscope. Immunohistochemistry demonstrated the positive expression of endothelial cell markers including CD31, CD34 and factor VIII, and the negative expression of CK, CK8/18, CK19, Glypican 3, CD 21 and CD 68, the Ki-67 proliferative index was 30%. Hepatic angiosarcoma was finally confirmed. The patient died of tumor metastasis 2 months after operation.

## Discussion and conclusions

Diffused hepatic angiosarcoma is an extremely rare malignant tumor characterized by aggressive proliferation and wide distribution of tumor cells. Despite the low incidence of hepatic angiosarcoma, it is the most common primarily malignant mesenchymal tumor of the liver in adults. It accounts for 2% of all primary hepatic malignancies [1, 6, 7]. The pathogenesis of hepatic angiosarcoma is still uncertain, exposure to polyvinyl chloride and arsenic can lead to a high incidence of hepatic angiosarcoma [8].

KMS, also known as Kasabach-Merritt phenomenon (KMP), is frequently found in children with kaposiform haemangioendothelioma or tufted angioma. A study designed by Ji Y confirmed that approximately 70% of patients diagnosed with kaposiform haemangioendothelioma showed KMP, lesions involving the trunk were more likely to have KMP than non-trunk lesions, and they found that age at discovery of the tumor lesion, morphology and tumor size were independent risk factors for KMP [9]. KMP is considered as a rare complication of vascular tumors [10]. Liver failure which is frequently caused by pharmacological toxicity and viral hepatitis is seldom induced by primary liver tumors including hepatic angiosarcoma.

Despite the hypothesis that KMP is exclusively limited to these two vascular formations in paediatric populations [11], there have been several case reports about hepatic angiosarcoma with KMS in adults recently [12]. KMS is characterized by anemia, thrombocytopenia, prolonged prothrombin time, hypofibrinogenemia, and obviously increased fibrinogen degradation product and D-dimer. Based on the blood test results, the patient was diagnosed with KMS.

Liver hemangioma was diagnosed mistakenly before operation due to the insufficiency of typical imaging features for angiosarcoma. Diagnostic indicators are difficult to obtain for distinguishing angiosarcoma from hemangioma. Some studies suggested that there were several imaging features on contrast-enhanced CT: hepatic angiosarcoma should be highly suspected if non-peripheral enhancement and arteriovenous short circuit were observed; while the radiological features of hepatic hemangioma was characterized by peripheral nodular enhancement, arteriovenous short circuit was scarce [3, 13, 14]. Unfortunately, it was difficult to get the correct diagnosis by observing patient’s imaging before operation.

Radical resection followed by targeted therapy is the most effective treatment strategy for hepatic angiosarcoma [15]. Less than 20% of patients were suitable for radical hepatectomy due to its aggressive behavior and multifocal distribution [1]. Liver transplantation is not recommended since no advantages for survival in those patients who received transplantation incidentally or with therapeutic intentions [16]. Median survival after liver transplantation is less than 7 months according to European Liver Transplant Registry [16]. Liver transplantation was conducted for this patient on account of liver failure and incorrect diagnosis. Standard adjuvant chemotherapy regimens have not been established for the disease till now.

Because of highly aggressive behavior, low radical resection rate and insensitivity to chemoradiotherapy, hepatic angiosarcoma has a very high relapse rate and short survival time. The vast majority of patients have a mean survival time of less than 6 months if no treatment is provided [6, 15]. Even if treatment is conducted, only 3% of patients have the survival time of more than 2 years [17]. Groeschl et al. [18] reported that the median overall survival time of patients with hepatic angiosarcoma was only 1 month, and survival time can be prolonged to 6 months if patients received radical resection. Patient died of lung and brain metastasis 2 month after liver transplantation in this report.

In conclusion, hepatic angiosarcoma is a rare malignant tumor with very poor prognosis, it can result in KMS and liver failure. Radical resection followed by targeted therapy may be the most effective treatment strategy. Liver transplantation should not be considered in hepatic angiosarcoma regardless of the reason.

## Data Availability

The datasets used and/or analyzed in the current study are available from the corresponding author on reasonable request.

## Abbreviations

CT: Computed Tomography
DCD: Donation after circulatory death
INR: International Normalized Ratio
KMP: Kasabach-Merritt phenomenon
KMS: Kasabach-Merritt syndrome
